# Integrating social media-based community of inquiry with theory of planned behavior to promote equitable educational intentions among pre-service teachers in Gilgit Baltistan, Pakistan

**DOI:** 10.3389/fpsyg.2023.1150421

**Published:** 2024-04-16

**Authors:** Imdad Ullah, Muhammad Zaheer Asghar, Elena Barbera, Meltem Cimen

**Affiliations:** ^1^Department of Education, Karakoram International University, Gilgit, Pakistan; ^2^Department of Education, University of Helsinki, Helsinki, Finland; ^3^Learning and Educational Technology (LET Lab), Faculty of Education and Psychology, Oulu University, Oulu, Finland; ^4^Faculty of Education Psychology, Open University of Catalonia, Barcelona, Spain; ^5^Department of Music Education, Dokuz Eylul University, Izmir, Türkiye

**Keywords:** community of inquiry, pre-service teachers, theory of planned behavior, social media, social justice

## Abstract

An equitable education system is essential for all students to acquire the knowledge and skills necessary to become productive members of society. Pre-service teachers in education play a vital role in fostering equitable educational practices. This study aimed to measure the association between the social media-based community of inquiry and pre-service teachers’ intentions toward social justice and equity in education. It focused on pre-service teachers enrolled in the education departments of universities in Gilgit Baltistan (GB), Pakistan. Census sampling was used to include all students enrolled in teacher education departments across universities in GB. The research utilized a multi-wave survey design, beginning with a baseline survey to assess pre-service teachers’ presence on social media. This information guided the design of a community of inquiry on social media centered on the theme of social justice and equity in education. After 4 months, a second survey was conducted to measure the association between the community of inquiry and pre-service teachers’ intentions toward social justice and equity. For data analysis, the study employed the partial least squares-consistent structural equation modeling (PLSc-SEM) approach. The novelty of the study lies in integrating the community of inquiry framework with the theory of planned behavior. We found a significant and positive association between the social media-based community of inquiry and pre-service teachers’ attitudes, subjective norms, and perceived behavioral control regarding their intentions to implement social justice and equity in education. These findings hold the potential for developing prospective teachers and educational leadership with a strong focus on equity. Future research could explore creating a community of inquiry for pre-service teachers to enhance their mindset and skills for inclusive education. This aligns with the broader objective of achieving Sustainable Development Goal 4 (SDG 4) on fostering a more inclusive and equitable educational environment.

## 1. Introduction

Sustainable Development Goal 4 (SDG 4) is one of the 17 SDGs adopted by the UN member states in 2015 as part of the 2030 Agenda for Sustainable Development (Anane-Simon and Olusegun Atiku, 2023). SDG 4 is focused on ensuring inclusive, equitable, and quality education for all and promoting lifelong learning opportunities (Rasoolimanesh et al., 2020). Pakistan faces significant challenges in achieving SDG 4 due to a notable number of out-of-school children, particularly in regions like Gilgit Baltistan (ASER, 2014). The Gilgit Baltistan (GB) province is characterized by its diverse population, with variations in ethnicity, social class, political affiliations, and cultural recognition. Despite acknowledging education as a basic right and the government prioritizing it, stratified education exacerbates disparities among different groups, leading to gender disparities in enrollment, high repetition and dropout rates in class five, and inadequate school facilities (Bursztyn and Drummond, 2014). In this context, the quality of teacher education significantly affects the educational system, with many teachers lacking professional training and higher qualifications (ASER, 2019).

Ensuring social justice and equity in education is paramount in addressing these disparities. The concept of social equity and justice has deep historical roots, emerging from religious and theological thinking (Kraynak, 2018). Rawls (1971) study on the ‘Theory of Justice’ catalyzed discussions on social injustices and inequities, leading to policy changes aimed at addressing these issues. Access to education is a fundamental aspect of social justice, emphasizing equality and fairness in educational opportunities (Ullah, 2013). However, formulating equity-related policies alone is inadequate; thus, effective implementation of these policies is crucial (Abdullah and Chaudhry, 2018). Effective teacher education and training are essential to develop human resources capable of implementing social justice-based equitable educational policies.

Pre-service teachers are students who are enrolled in teaching programs under the supervision of university teachers to obtain a degree in education. According to Menlah (2013), pre-service teachers are students pursuing a bachelor’s or master’s degree in education or a relevant field who have not yet completed the program. Universities must equip pre-service teachers with the mindset and latest skills for effective teaching in schools. Universities must train pre-service teachers to enhance their job performance (Griffiths et al., 2021). Pre-service teachers are the future practitioners who can help address the issues related to equity in education and social justice in remote areas such as Gilgit Baltistan. The intention of pre-service teachers to engage with social justice practices in schools is influenced by their attitudes, subjective norms, and perceived behavior control (Martin et al., 2021). Implementing social justice practices at the school and classroom levels is crucial, and teachers play a vital role in the front line of policy implementation (Pasha et al., 2021).

The prevalence of mobile phones and internet services in the region offers opportunities to leverage social media for the professional development of teachers in Gilgit Baltistan (ASER, 2019). Social media, as a group of applications facilitating social interaction and information sharing, can play a significant role in promoting equitable educational intentions among pre-service teachers (Majchrzak et al., 2013). Leveraging technology and social media can potentially bridge the gaps and promote equitable educational practices by enhancing access to resources and creating social connectivity (Popescu and Badea, 2020). The online community of inquiry (COI), a framework designed for purposeful e-learning communities, provides an effective platform for asynchronous debates and discussions, fostering a community of learners (Garrison, 2015). A community learning model is an effective form of online program where teachers connect with their instructors and also connect and interact with their peers. Learning occurs in a group through discussion. The instructor plays the role of a facilitator and allows open discussion. Participants discuss and reach any conclusion (Shaha et al., 2015).

Previous studies have shown that research related to online learning, social media-based learning, COI development, and pre-service teachers training for inclusive education is available in different segments. For example, previous research is focused mostly on e-learning or M-learning among higher education students (Qureshi et al., 2012). There are also studies that highlight the use of e-learning and other online learning sources, such as social media, for learning among teachers (Yu et al., 2021; Asghar et al., 2022). Researchers have also explored the formation of a COI in e-learning environments (Oyarzun et al., 2021), social media environments (Solmaz, 2016; Nazir and Brouwer, 2019; Popescu and Badea, 2020), and blended learning (Stenbom, 2018). Evidence is available on pre-service teachers’ training for inclusive education in face-to-face learning setups utilizing the theory of planned behavior (Yan and Sin, 2014; Pasha et al., 2021). However, fewer studies have highlighted the role of a community of inquiry in producing certain behavior. To the best of our knowledge, this study is the first of its kind to explore the role of a social media-based community of inquiry in promoting pre-service teachers’ intentions toward equitable education in remote areas such as Gilgit Baltistan. In this study, we aim to contribute valuable insights into utilizing technology to enhance equity in education and develop human resources to foster a more inclusive learning environment in the region. This study aimed to answer the question: “What is the role of the social media-based community of inquiry in enhancing pre-service teachers’ intentions toward equity in education?”

Our study contributes to previous literature in several ways. First, it provides evidence of using social media to develop a community of inquiry among pre-service teachers in remote areas of a developing country. Second, this study addresses the formation of pre-service teachers’ intentions for equitable educational intentions. Third, it adopts a theoretically novel approach to integrate the community of inquiry theory with the theory of planned behavior. Fourth, it is a methodological addition to the study’s use of partial least square structural equation modeling for the theoretical exploration of a social media-based community of inquiry and its link with the theory of planned behavior.

This study would be useful for higher educational institutions, the Ministry of Education, and teacher training institutes to formulate their policies and design their training programs to use available emerging technologies, such as social media, to effectively train pre-service teachers to achieve SDG 4.

The first section of this article provides the background, rationale, statement of the problem, and significance of the study. The second section presents the theoretical framework of the study. The third section outlines the conceptual framework and hypothesis formation of the study. The fourth section details the research methodology, population, sample, and sampling procedures. The “Data analysis” section presents data analysis and findings. The “Discussion” section consists of the discussion, conclusion, and implications.

## 2. Theoretical frameworks

In this study, we combined the community of inquiry (COI) framework and the theory of planned behavior (TPB) to investigate the relationship between COI and pre-service teachers’ intentions toward equity in education. This study is based on the theoretical framework of the following:

1. Social justice
2. Theory of planned behavior
3. Community of inquiry

First, we used the concept of social justice to explain equitable education. The concept of social justice was developed and introduced in religious and theological thinking (Kraynak, 2018). John Rawls’ *Theory of Justice* triggered debates and discussions in the literature (Rawls, 1971), and social injustice and social inequities came to be highlighted and recognized as social issues. Public policies and administrations started to address these social issues, leading to the formulation of better policies and implementation procedures. Over time, social equity and social justice encompassed all spheres of life. Social justice is defined as a fair distribution of resources, power, privileges, and obligations without discrimination based on socioeconomic status, gender, race, or religion. According to this definition, fairness is the essence of justice in society (Prilleltensky, 2013). According to Rawls (1971), justice is not just a product or a system of institutions, but it also includes judgments, decisions, attitudes, and behaviors. These decisions, judgments, and behaviors may be just or unjust, and the essence of social justice depends on how society promotes and distributes rights and responsibilities (Rawls, 1971). Social justice, inclusion, and equity are terms used with the same meaning in literature and face the same problems and issues in their propagation (Ryan, 2017).

Equity is a state or condition of inclusion and fairness in any type of affair and treatment of all people in society. Treating people in the same way while ignoring their needs and individual differences does not represent equity. In this regard, equity is different from equality, where all people are treated the same way. Ryan (2017) elaborated on the difference between equity and equality, pointing out that inequalities may co-exist with equality due to the disregard for individual needs and differences. True equity involves considering the needs and differences of people in the distribution and recognition process. Social justice and equity are commonly used with the same meaning and concept, and inclusion is also related to equity and social justice (Ryan, 2017).

Second, we used the theory of planned behavior (Ajzen, 1985) to measure pre-service teachers’ intentions toward equitable education. The TPB is mainly used to predict or explain behavioral patterns in human beings under certain circumstances (Ajzen, 2020). This theory posits that behavioral intentions are influenced by attitude, subjective norm, and perceived behavioral control, while perceived behavioral control and behavioral intention directly affect actual behavior (Hill et al., 1977). Attitude, subjective norms, and perceived behavioral control are the basic constructs of TPB, and they play a vital role in predicting behavioral intention (Fishbein and Ajzen, 2011). Attitude represents a state of readiness to respond to any situation or object and is measured through the specific behavior of an individual, which may be positive or negative (Schmidt et al., 2022). While a positive attitude is logically antecedent to a positive intention, the attitude is not the only factor shaping behavior. Subjective norms, as the second construct of TPB, represent the social factors and pressures faced by an individual while performing or avoiding a behavior (Ajzen, 2020). Perceived behavioral control refers to an individual’s perception of their ability to perform a behavior while considering the ease and difficulty of performing that behavior (Asghar et al., 2019), while intentions are defined as a person’s behavior to perform certain actions (Knabe, 2009). This study operationalized pre-service teachers’ intentions to promote equitable education in Gilgit Baltistan.

Third, this study operationalized the community of inquiry framework in the context of pre-service teachers’ participation in social media-based learning environments. The COI is a widely used and referenced framework for online and blended learning (Garrison and Akyol, 2015). It is designed for purposeful e-learning communities and computer-based conferencing, utilizing asynchronous discussions to connect learners and create a community of learners. COI offers an effective collaborative and online learning environment where knowledge is co-constructed through social presence, cognitive presence, and teaching presence. Cognitive presence involves the learner’s ability to construct, create, and derive meaning by reflecting in a community of inquiry (Akyol and Garrison, 2009). It encompasses discourse, inquiry, and resolution of the content. Social presence plays a mediating role between cognitive and teaching presences by synchronizing interactions between teachers, students, content, and media tools within an online experience. Social presence depends on the ability of learners to communicate in a trustworthy environment and develop strong relationships with the participants in the community (Richardson and Lowenthal, 2017). Teaching presence refers to designing course content, facilitating while delivering the content, and providing instructional directions for meaningful learning. It has three principles that support social presence and cognitive presence: content design, facilitation, and direction (Garrison, 2015). Connecting pre-service teachers through online COI would be an innovative and effective mode of professional development.

## 3. Hypothesis development and conceptual framework

The theoretical foundations of the study provided the basis to develop a relationship between the COI and TPB factors. It led to the development of hypotheses and the conceptual framework of the study, as described in this section.

### 3.1. Social media-based COI and the theory of planned behavior

As discussed in the previous section, the theory of planned behavior comprises three major components, attitude, subjective norms, and perceived behavioral control, which influence an individual’s intentions toward certain behaviors. Previous studies have shown a positive association between participation in social media environments and TPB components in various contexts. For instance, Latif and Calicioglu (2020) found that the use of social media for advertisement was linked to individuals’ preferences and opinions about certain brands, concepts, and ideas. Additionally, Duffett (2017) demonstrated that young individuals who spend more time on social media had a favorable attitude toward advertised products, indicating that social media participation influenced the attitudes of the younger generation concerning specific ideas. Furthermore, research in the area of teacher education, such as that conducted by Blasco-Serrano et al. (2022), showed that pre-service teachers’ participation in online learning environments positively influenced their attitude toward inclusive education.

Previous studies also provide evidence that participation in social media environments connects users with like-minded subjective norms, such as friends, peers, and colleagues. For example, the study “The impact of social media on green products purchase motivation and intention” by Pop et al. (2020) found a positive relationship between social media participation and the inclination of subjective norms toward green products. Similarly, Main et al. (2016) showed that teachers’ participation in virtual environments was linked to subjective norms that encourage them to embrace inclusive education. Similarly, social media use was found to have a positive relationship with subjective norms, perceived behavioral control, and the purchase intention of customers (Sun and Xing, 2022). In their study, Liu et al. (2022) concluded that social media played an essential role in increasing the perceived behavioral control (PBC) of its users concerning certain behaviors.

Overall, individuals’ participation in social media environments has a significant positive association with their attitude, subjective norms, and perceived behavioral control, which ultimately influences their intentions with regard to certain behaviors (Ringim and Reni, 2019). Therefore, we propose hypotheses linking the social media-based community of inquiry with the components of the theory of planned behavior in the context of pre-service teachers’ behavior toward equitable education as follows:

*Hypothesis 1.1*: Pre-service teachers’ participation in a social media-based community of inquiry is positively associated with their attitude toward social justice and equity in education.

*Hypothesis 1.2*: Pre-service teachers’ participation in a social media-based community of inquiry is positively associated with their subjective norms toward social justice and equity in education.

*Hypothesis 1.3*: Pre-service teachers’ participation in a social media-based community of inquiry is positively associated with their perceived behavioral control toward social justice and equity in education.

#### 3.1.1. Attitude, subjective norms, and perceived behavioral control and pre-service teachers’ intentions

Various studies consider teachers’ positive attitudes with respect to inclusion and social justice an essential requirement for the successful implementation of equity in education (De Boer et al., 2011; Van Mieghem et al., 2020). Researchers have provided evidence that teachers’ attitudes play a crucial role in determining their intentions toward the adoption of social justice in their educational practices (Malak et al., 2018; Knauder and Koschmieder, 2019).

Subjective norms, in the context of social justice and equity in education, refer to the extent to which a teacher’s intention to implement equitable educational practices is influenced by the behavior and actions of significant others, such as school principals, peers, colleagues, and parents of students (Gilor and Katz, 2019). Perceived norms, on the other hand, have been found to have a comparatively weaker influence on an individual’s intentions when compared to attitudes and perceived behavioral control (Armitage and Conner, 2001). However, researchers have found that subjective norms influence the attitude and perceived behavioral control (Karahanna et al., 2011; Martin et al., 2021) of teachers regarding the implementation of inclusion and equity in education. Subjective norms of teachers can influence their willingness to implement equitable educational practices, and the level of attitude that teachers attach to their opinions plays a crucial role in this matter (Ajzen, 2020).

Perceived behavioral control is closely related to self-efficacy — a concept described by Bandura (2001) that has been more commonly studied compared to perceived behavioral control when measuring its influence on a person’s intent (Knabe, 2009). Previous studies have indicated that teachers’ intentions toward the implementation of inclusive and social justice-based educational practices are significantly predicted by both their self-efficacy and collective efficacy (Malak et al., 2018). Based on this discussion, we propose the following hypotheses:

*Hypothesis 2.1*: The attitude of pre-service teachers has a positive association with their intentions toward social justice and equity in education.

*Hypothesis 2.2*: The perceived behavioral control of pre-service teachers has a positive association with their intentions toward social justice and equity in education.

*Hypothesis 2.3*: Subjective norms of pre-service teachers have a positive association with their attitude toward social justice and equity in education.

*Hypothesis 2.4*: Subjective norms of pre-service teachers have a positive association with their perceived behavioral control toward social justice and equity in education.

### 3.2. Conceptual framework

**Figure 1:**
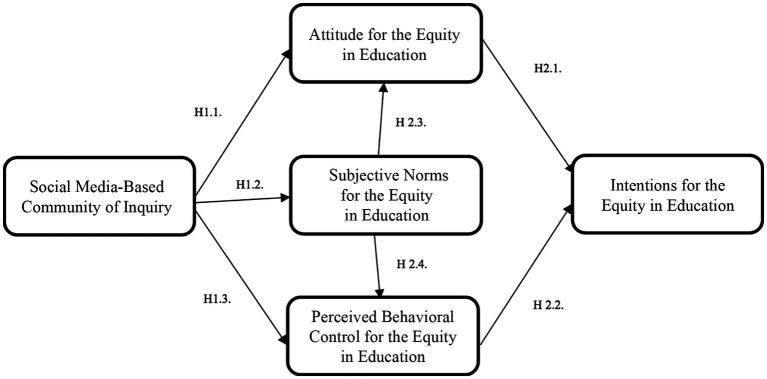
COI and TPB for social justice and equity in education.

In this study, we combined the community of inquiry and the theory of planned behavior. The social media-based COI was operationalized as a single construct, and it served as an exogenous variable for the three TPB components: attitude, subjective norms, and perceived behavior control. These three components, in turn, were considered exogenous constructs for the endogenous construct of pre-service teachers’ intentions toward equity in education. The conceptual framework explored the relationship between pre-service teachers’ participation in a social media-based COI and their attitudes, subjective norms, and perceived behavioral control toward social justice and equity in education (hypotheses 1.1, 1.2, and 1.3). Additionally, it investigated the associations between pre-service teachers’ attitudes, subjective norms, and perceived behavioral control with their intentions concerning social justice and equity in education (hypotheses 2.1, 2.2, 2.3, and 2.4). The arrows in Figure 1 represent the positive associations between the variables indicated in the hypotheses. The conceptual framework is based on the hypotheses, as shown in Figure 1.

## 4. Research method

### 4.1. Research approach

This study employed a quantitative research approach and a multi-phase survey design. The decision to use a survey design was based on three reasons: first, the need for a large amount of data to generalize the results; second, the focus on individual perceptions, particularly pre-service teachers’ presence in the COI and its association with their intentions, which necessitated self-reported data; and third, the convenience of data collection. Consequently, we conducted the initial survey at the beginning of the study to assess pre-service teachers’ presence in the COI through social media. The survey findings assisted teacher education institutions in Gilgit Baltistan to develop education programs through social media centered around the theme of equity in education during the COVID-19 lockdown. After 4 months, a second survey was conducted to measure the association between the COI and pre-service teachers’ intentions toward social justice and equity.

### 4.2. Population and sampling

There are two universities in Gilgit Baltistan. The unit of analysis for this study was pre-service teachers enrolled in the education departments of these two universities. Ethical approval was obtained from a local university to conduct the study. An email invitation was sent to the heads of the education departments to invite students for voluntary participation. The students provided their consent to participate voluntarily in the study. There were a total of 350 students enrolled in different programs at the two universities, with 200 students in one university and 150 in the other. A census sample was taken, including all 350 pre-service teachers, to conduct the baseline survey in both universities.

After data screening, including the removal of incomplete forms, missing values, and outliers, a total of 225 complete responses were included in the final data analysis, which represented 64.29% of a total population of 350. This sample size was justified based on the anticipated effect size of 0.5, the desired statistical power level of 0.05, the number of latent variables (5), the number of observed variables (32), and the probability level of 0.05. This sample size met the criteria for detecting significant effects and maintained an appropriate ratio of participants to observed variables, achieving a desired level of statistical confidence (Daniel, 2023).

Table 1 provides information on various measures, including categories, frequencies, and percentages. In terms of gender distribution, approximately 82.66% of the total respondents were men, while women constituted the remaining 17.33%. Regarding location, approximately 24.44% of the respondents were from rural areas, while a majority (75.55%) were from urban areas. In terms of qualification, approximately 68.88% of the individuals were enrolled in an undergraduate program, and a significant majority of 31.11% were enrolled in a graduate program (see Table 1).

**Table 1:** Demographic information.

Measures	Categories	Frequencies (*f*)	Percentage (*%*)
Gender	Women	39	17.33
	Men	186	82.66
	Total	225	100
Location	Rural	55	24.44
	Urban	170	75.55
	Total	225	100
Qualification	Undergraduate	155	68.88
	Graduate	70	31.11
	Total	225	100

### 4.3. Constructs measurement

#### 4.3.1. Attitude toward social justice and equity in education (SJA)

Seven items related to the social justice attitude were adapted from the social justice scale developed by Torres-Harding et al. (2012) for this study. Students were asked to assess their attitudes regarding social justice and equity. The sample question items were: “I believe that it is important to make sure that all individuals and groups have a chance to speak and be heard, especially those from traditionally ignored or marginalized groups”; “I believe that it is important to allow individuals and groups to define and describe their problems, experiences, and goals in their terms”; “I believe that it is important to help individuals and groups pursue their chosen goals in life”; and “I believe that it is important to act for social justice.” Each item was rated on a 5-point Likert scale ranging from 1 = Totally Disagree to 5 = Totally Agree. The sum of the answers was divided by 5 to obtain the mean value of the construct. The mean value obtained was 3.78, which reflected the agreement of the respondents on this construct. The Cronbach’s alpha reliability of the attitude toward social justice and equity in education (SJA) construct was found to be α = 0.848, indicating the consistency of the construct.

#### 4.3.2. Intentions toward social justice and equity in education (SJI)

Four items related to pre-service teachers’ intentions toward social justice and equity in education were adapted from the social justice scale developed by Torres-Harding et al. (2012). The sample question items were as follows: “In the future, I will do my best to ensure that all individuals and groups have a chance to speak and be heard”; “In the future, I intend to talk with others about social power inequalities, social injustices, and the impact of social forces on health and well-being”; and “In the future, I intend to engage in activities that will promote social justice.” Each item was rated on a 5-point Likert scale ranging from 1 = Totally Disagree to 5 = Totally Agree. The sum of the answers was divided by 5 to get the mean value of the construct. The mean value obtained was 3.59, indicating an agreement of the respondents on this construct. The Cronbach’s alpha reliability of the intentions toward social justice and equity in education (SJI) construct was found to be α = 0.775, indicating the consistency of the construct.

#### 4.3.3. Perceived behavioral control toward social justice and equity in education (SJPB)

Five items related to pre-service teachers’ perceived behavioral control toward social justice and equity in education were adapted from the social justice scale developed by Torres-Harding et al. (2012). The sample question items were as follows: “I am confident that I can have a positive impact on others’ lives”; “I am certain that I possess the ability to work with individuals and groups in ways that are empowering”; and “If I choose to do so, I am capable of influencing others to promote fairness and equality.” Each item was rated on a 5-point Likert scale ranging from 1 = Totally Disagree to 5 = Totally Agree. The sum of the answers was divided by 5 to get the mean value of the construct. The mean value obtained was 4.05, indicating an agreement of the respondents on this construct. The Cronbach’s alpha reliability of the perceived behavioral control toward social justice and equity in education (SJPB) construct was found to be α = 0.755, indicating the consistency of the construct.

#### 4.3.4. Subjective norms toward social justice and equity in education (SJSN)

Four items related to pre-service teachers’ subjective norms toward social justice and equity in education were adapted from the social justice scale developed by Torres-Harding et al. (2012). The sample question items were as follows: “Other people around me are engaged in activities that address social injustices”; “Other people around me feel that it is important to engage in dialog around social injustices”; and “Other people around me are supportive of efforts that promote social justice.” Each item was rated on a 5-point Likert scale ranging from 1 = Totally Disagree to 5 = Totally Agree. The sum of the answers was divided by 5 to obtain the mean value of the construct. The mean value obtained was 3.77, indicating an agreement of the respondents on this construct. The Cronbach’s alpha reliability of the subjective norms toward social justice and equity in education (SJSN) construct was found to be α = 0.817, indicating the consistency of the construct.

#### 4.3.5. Community of inquiry scale

The COI instrument developed by Garrison (2015) was adapted to measure social presence, cognitive presence, and teaching presence. The instrument finalized for this study consisted of 12 items divided into three sub-constructs.

#### 4.3.6. Cognitive presence

Four items related to pre-service teachers’ cognitive presence in the social media-based COI were adapted from the COI scale developed by Garrison (2015). The sample question items were as follows: “Online discussions were valuable in helping me to appreciate different perspectives”; “Combining new information helped me to answer questions raised in social media group/page activities”; and “Learning activities helped me to construct explanations and solutions.” Each item was rated on a 5-point Likert scale ranging from 1 = Totally Disagree to 5 = Totally Agree. The sum of the answers was divided by 5 to obtain the mean value of the construct. The mean value obtained was 3.43, indicating an agreement of the respondents on this construct. The Cronbach’s alpha reliability of the cognitive presence construct was found to be α = 0.897, indicating the consistency of the construct.

#### 4.3.7. Social presence

Four items related to pre-service teachers’ social presence in the social media-based COI were adapted from the COI scale developed by Garrison (2015). The sample question items were as follows: “I felt comfortable participation in group discussion on the topic of social justice and equity in education”; “I felt comfortable on social media while interacting with other participants on the topics of social justice and equity in education”; and “I felt comfortable in disagreeing on some topics with group/page participants while still maintaining a sense of trust.” Each item was rated on a 5-point Likert scale ranging from 1 = Totally Disagree to 5 = Totally Agree. The sum of the answers was divided by 5 to get the mean value of the construct. The mean value obtained was 4.21, indicating the agreement of the respondents on this construct. The Cronbach’s alpha reliability of the social presence construct was found to be α = 0.876, indicating the consistency of the construct.

#### 4.3.8. Teaching presence

Four items related to pre-service teachers’ teaching presence in the social media-based COI were adapted from the COI scale developed by Garrison (2015). The sample question items were as follows: “The instructor took action to reinforce the development of a sense of community on educational issues among their followers”; “The instructor helped to focus discussions on relevant issues in a way that helped me to learn”; and “The instructor provided feedback/comments that helped me to understand my strength and weaknesses.” Each item was rated on a 5-point Likert scale ranging from 1 = Totally Disagree to 5 = Totally Agree. The sum of the answers was divided by 5 to obtain the mean value of the construct. The mean value obtained was 4.06, indicating the agreement of the respondents on this construct. The Cronbach’s alpha reliability of the social presence construct was found to be α = 0.911, indicating the consistency of the construct.

## 5. Data analysis

The partial least square-consistence structural equation model (PLSc-SEM) was used to measure the association of the community of inquiry with the components of the theory of planned behavior for social justice and equity intentions of the pre-service teachers. The conceptual model of the study was complex, so SmartPLS 3.2.8 was employed to explore the relationships among the variables (Hair, 2017). Since the COI had three constructs — cognitive presence, social presence, and teaching presence — a second-order factor analysis was performed to combine these three constructs. In the first step, we analyzed the outer model measurement. Secondly, we checked convergent and discriminant validity. Subsequently, we analyzed model fit, collinearity, and R-square (Asghar et al., 2023).

### 5.1. Measurement model evaluation

The reliability of the outer model constructs was assessed following the guidelines of Ho (2013). Constructs with alpha scores above the threshold of 0.6 were considered acceptable, as suggested by Hair et al. (2014). All items showed factor loadings above 0.7. Convergent validity was measured using the average variance extracted (AVE) values, and the threshold value of 0.5 for validity was met (Fornell and Larcker, 1981). The standard set for composite reliability (CR) with a threshold of 0.7 was also met. For the confirmatory factor analysis (CFA) of the primary factors, a first-order analysis was performed. The reliability measures (α > 0.7, rho >0.7), convergent validity (AVE > 0.5), and composite reliability (CR > 0.7) for all factors were found to be satisfactory (Table 2).

**Table 2:** Reliability and validity of first-order constructs.

Constructs	Item	Loading	Cronbach’s alpha	rho_A	Composite reliability	Average variance extracted (AVE)
SJA	SJA1	0.74	0.848	0.853	0.884	0.522
	SJA2	0.762				
	SJA3	0.723				
	SJA4	0.694				
	SJA5	0.678				
	SJA6	0.705				
	SJA7	0.754				
SJI	SJBI1	0.715	0.775	0.78	0.847	0.526
	SJBI2	0.763				
	SJBI3	0.817				
	SJBI4	0.739				
SJPB	SJPB1	0.697	0.755	0.765	0.845	0.577
	SJPB2	0.741				
	SJPB3	0.747				
	SJPB4	0.695				
	SJPB5	0.743				
SJSN	SJSN1	0.822	0.817	0.82	0.879	0.646
	SJSN2	0.826				
	SJSN3	0.806				
	SJSN4	0.759				
TP	TP1	0.89	0.911	0.914	0.938	0.79
	TP2	0.865				
	TP3	0.914				
	TP4	0.887				
CP	CP1	0.871	0.897	0.897	0.929	0.765
	CP2	0.881				
	CP3	0.851				
	CP4	0.894				
SP	SP1	0.846	0.876	0.877	0.915	0.728
	SP2	0.84				
	SP3	0.864				
	SP4	0.863				

SJA, Attitude toward social justice and equity in education; SJI, Intentions toward social justice and equity in education; SJPB, Perceived behavioral control toward social justice and equity in education; SJSN, Subjective norms toward social justice and equity in education; CP, Cognitive presence; SP, Social presence; TP, Teaching presence.

We used the heterotrait–monotrait (HTMT) correlation ratios to measure the discriminant validity. According to Henseler et al. (2015), an HTMT value below 1 is considered acceptable. In the current study, all the constructs had HTMT values below 0.9, which is considered satisfactory (Table 3).

**Table 3:** Heterotrait–monotrait correlation ratios.

	CP	SJA	SJPB	SJI	SP	SJSN	TP
CP							
SJA	0.366						
SJPB	0.419	0.821					
SJI	0.283	0.744	0.829				
SP	0.961	0.346	0.416	0.256			
SJSN	0.263	0.579	0.716	0.604	0.276		
TP	0.851	0.354	0.352	0.221	0.88	0.232	

SJA, Attitude toward social justice and equity in education; SJI, Intentions toward social justice and equity in education; SJPB, Perceived behavioral control toward social justice and equity in education; SJSN, Subjective norms toward social justice and equity in education; CP, Cognitive presence; SP, Social presence; TP, Teaching presence.

### 5.2. Second-order factor

Second-order analysis was conducted for the CFA of the constructs of COI. The reliability values (α > 0.7, rho >0.7), AVE (> 0.5), and CR (> 0.7) were found to be satisfactory for all factors (Hundleby and Nunnally, 1968). The alpha score above the threshold of 0.6 for constructs was considered acceptable. All items had factor loadings above 0.4. Convergent validity was measured using AVE values, and the threshold value of 0.5 for validity was met (Fornell and Larcker, 1981). The set standard for CR with a threshold of 0.7 was also met, as shown in Table 4.

**Table 4:** Reliability of second-order factor.

	Constructs	Loading>0.4	Cronbach’s alpha	rho_A	Composite reliability	Average variance extracted (AVE)
COI	CP	0.971	0.963	0.967	0.976	0.932
	SP	0.97				
	TP	0.954				

CP: Cognitive presence; SP: Social presence; TP: Teaching presence; COI, Community of inquiry.

Since the HTMT values for social presence (SP) and cognitive presence (CP) were above 0.95, it was necessary to conduct a second-order factor analysis of the COI, which includes CP, SP, and TP. All these dimensions showed a satisfactory level of HTMT ratio, which was found to be below 1, as shown in Table 5.

**Table 5:** Second-order HTMT.

	COI	SJA	SJPB	SJI	SJN
COI					
SJA	0.379				
SJPB	0.426	0.821			
SJI	0.286	0.744	0.829		
SJSN	0.269	0.579	0.716	0.604	

SJA, Attitude toward social justice and equity in education; SJI, Intentions toward social justice and equity in education; SJPB, Perceived behavioral control toward social justice and equity in education; SJSN, Subjective norms toward social justice and equity in education; COI, Community of inquiry.

### 5.3. Structural model measurement

After assessing the outer model, the validity and reliability of all constructs were found to be appropriate. The inner model evaluation comprised R-square, f-square, VIF, and goodness of fit measures (Henseler et al., 2015).

### 5.4. R-square

R-square measures the goodness of fit of the model and explains the variance in dependent and independent variables. The value of R-square should be above the threshold value of 0.1. In Table 6, the values of all four constructs are higher than the threshold and are accepted.

**Table 6:** R-square.

	*R* square	*R* square adjusted
SJA	0.296	0.287
SJPB	0.383	0.375
SJI	0.47	0.463
SJSN	0.061	0.055

SJA, Attitude toward social justice and equity in education; SJI, Intentions toward social justice and equity in education; SJPB, Perceived behavioral control toward social justice and equity in education; SJSN, Subjective norms toward social justice and equity in education.

### 5.5. Effect size

F-Square measures the effect of the independent variable on the dependent variable. The value of the f-square must be higher than the threshold value of 0.02 (Cohen, 1988). If the value of the f-square is below 0.02, the effect size would be considered weak. In Table 7, all constructs have values higher than the threshold value, indicating a strong effect size.

**Table 7:** *F*-square values.

	COI	SJA	SJPB	SJI	SJN
COI		0.078	0.086		0.065
SJA				0.105	
SJPB				0.192	
SJI					
SJSN		0.248	0.404		

SJA, Attitude toward social justice and equity in education; SJI, Intentions toward social justice and equity in education; SJPB, Perceived behavioral control toward social justice and equity in education; SJSN, Subjective norms toward social justice and equity in education; COI, Community of inquiry.

### 5.6. Variance inflation factor VIF

The inner VIF value of all constructs was below the threshold value of 0.5 (Salmerón et al., 2018), indicating that there was no issue of multicollinearity among the constructs. This is shown in Table 8.

**Table 8:** VIF values.

	COI	SJA	SJPB	SJI	SJN
COI		1.065	1.065		1
SJA				1.818	
SJPB				1.818	
SJI					
SJSN		1.065	1.065		

SJA, Attitude toward social justice and equity in education; SJI, Intentions toward social justice and equity in education; SJPB, Perceived behavioral control toward social justice and equity in education; SJSN, Subjective norms toward social justice and equity in education; COI, Community of inquiry.

### 5.7. The goodness of model Fit

To measure the model fitness, the standardized root mean residual (SRMR) was used. The SRMR value must be less than the threshold value of 0.8. In the current study, the SRMR value was 0.07, which is below the threshold value and accepted. According to Asghar et al. (2022), Hu and Bentler (1998), and Zhou et al. (2021), a path model with an SRMR value below 0.08 is considered to be a good fit. The normed fit index (NFI) values were above the threshold value of 0.8, as shown in Table 9.

**Table 9:** Model fit values.

	Saturated model	Estimated model
SRMR	0.07	0.097
d_ULS	1.366	2.617
d_G	0.548	0.617
Chi-Square	486.254	514.379
NFI	0.767	0.753

### 5.8. Redundancy analysis

We measured the predictive criterion accuracy with Geisser’s (1975) Q2 value. The Q2 value assesses the quality of the model. A cross-validity redundancy analysis yielded a value of Q2 (=1 – SSE/SSO) that is above the threshold value of 0, which is considered acceptable, as shown in Table 10.

**Table 10:** Cross validity redundancy analysis.

	SSO	SSE	*Q*^2^ (=1−SSE/SSO)
COI	471	471	
SJA	1,099	936.143	0.148
SJPB	785	634.647	0.192
SJI	628	465.006	0.26
SJSN	628	604.87	0.037

SJA, Attitude toward social justice and equity in education; SJI, Intentions toward social justice and equity in education; SJPB, Perceived behavioral control toward social justice and equity in education; SJSN, Subjective norms toward social justice and equity in education; COI, Community of inquiry.

### 5.9. Direct path analysis

The mean path β coefficient for regression values was used in PLS-SEM to test the hypotheses. Table 11 shows the coefficients (β) of the strength of the paths, while the T statistics and the value of p for each hypothesis reflect the probability of acceptance or rejection. All hypotheses were accepted as the value of p was below the significance level of 0.05, as shown in Table 11.

**Table 11:** Path analysis.

Hypothesis	Paths	*β*	T statistics (|O/STDEV|)	*p*-value	Status
H1.1.	COI - > SJA	0.241	3.704	0.000	Accepted
H1.2.	COI - > SJSN	0.244	3.371	0.001	Accepted
H1.3.	COI - > SJPB	0.238	4.032	0.000	Accepted
H2.1.	SJA - > SJI	0.314	3.804	0.000	Accepted
H2.2.	SJPB - > SJI	0.438	4.337	0.000	Accepted
H2.3.	SJSN - > SJA	0.434	6.185	0.000	Accepted
H2.4.	SJSN - > SJPB	0.521	9.724	0.000	Accepted

SJA, Attitude toward social justice and equity in education; SJI, Intentions toward social justice and equity in education; SJPB, Perceived behavioral control toward social justice and equity in education; SJSN, Subjective norms toward social justice and equity in education; COI, Community of inquiry.

**Figure 2:**
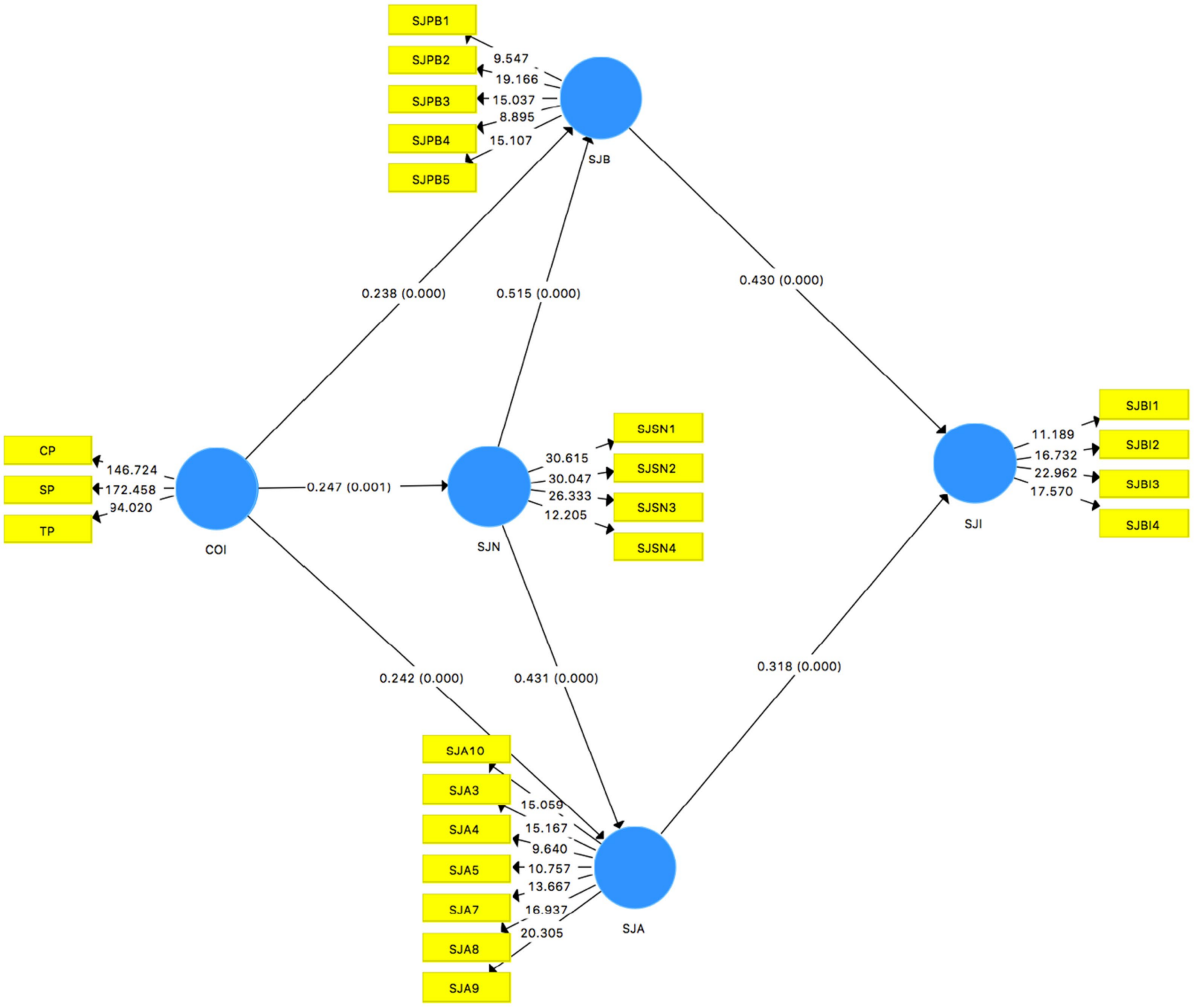
Direct relations.

Figure 2 shows the strength of association and the probability level among the constructs.

## 6. Discussions

The purpose of this study was to investigate the association between the social media-based community of inquiry and pre-service teachers’ intentions toward social justice and equity in education. To the best of our knowledge, this study is among the pioneering research endeavors that not only examine social media usage for educational purposes and the social, teaching, and cognitive presence of pre-service teachers but also integrate the COI with the theory of planned behavior to measure their intentions with regard to social justice and equity in education.

Path analysis was used to measure the association between COI constructs and TPB. Path analysis is a multiple regression statistical analysis used to evaluate the association between independent and dependent variables. We combined the three components of the COI, such as social presence, teaching presence, and cognitive presence, at second-order factor analysis. The association of social media-based COI with the three components of the theory of planned behavior was analyzed through direct path analysis. The results showed that an association exists between COI and the first construct attitude toward social justice and equity in education (SJA) of the theory of planned behavior (*β* = 0.241, *t* = 3.704, *p* < 0.001). Thus, Hypothesis 1.1 was accepted. These results are consistent with previous studies, such as in a study on advertisement through social media, where Latif and Calicioglu (2020) found that the use of social media for advertisement had a direct association with customer brand attitude. Another study (Blasco-Serrano et al., 2022) showed that pre-service teachers’ training through blended learning approaches enhanced their attitude in favor of inclusive education. The results of our study also endorsed that a formal social media-based community of inquiry contributed to enhancing pre-service teachers’ attitudes toward social justice and equity in education.

Our results showed a positive and significant association between social media-based COI and pre-service teachers’ subjective norms toward social justice and equity in education (SJSN) construct of the TPB, so Hypothesis 1.2 was also accepted (*β* = 0.244, *t* = 3.371, *p* = 0.001). The results of previous studies in different disciplines support the results of the current study. For instance, Pop et al. (2020) found a relationship between social media participation and the subjective norms of customers toward green cosmetics. Another research indicated that social media activities influence purchase intention, while subjective norms and perceived behavioral control play a mediating role (Sun and Xing, 2022). Meanwhile, research conducted in the Pakistani context showed that social media use enhanced pre-service teachers’ subjective norms for their professional development (Hashmi et al., 2021). Therefore, the finding of our study confirms that the social media-based community of inquiry is useful for linking subjective norms such as peers, class fellows, and colleagues of pre-service teachers to enhance their intentions toward social justice and equity in education.

The statistical results of the current study show the association between social media-based COI and perceived behavioral control toward social justice and equity in education (SJPB) construct of the TPB. Therefore, Hypothesis 1.3 was accepted (*β* = 0.238, *t* = 4.032, *p* < 0.001). Previous research also confirms the association between individuals’ participation in social media-based environments and its relation with subjective norms to enhance their intentions concerning certain behaviors. For example, a previous study indicated that social media has a significant association with customer attitude, subjective norms, and perceived behavioral control (Ringim and Reni, 2019). Another research showed the influence of social media-based learning and collaboration on pre-service teachers’ self-efficacy to enhance their academic performance (Liu et al., 2022). In our study, we established the association between the social media-based community of inquiry and the perceived behavioral control of the pre-service teachers for their intentions to implement equitable education.

The findings of this study supported Hypothesis 2.1, revealing a significant positive association between pre-service teachers’ attitude toward social justice and equity in education (SJA) construct and their intentions toward social justice and equity in education (SJI) constructs (*β* = 0.314, *t* = 3.804, *p* < 0.001), as indicated by previous research (De Boer et al., 2011; Van Mieghem et al., 2020). These results align with previous studies that emphasized the importance of teachers’ positive attitudes concerning inclusion and social justice in fostering the successful implementation of equity in education (De Boer et al., 2011; Van Mieghem et al., 2020). Other researchers have also provided evidence of the critical role played by teachers’ attitudes in shaping their intentions in embracing social justice in their educational practices (Malak et al., 2018; Knauder and Koschmieder, 2019). Thus, the current study’s outcomes further validate the significance of pre-service teachers’ attitudes toward social justice as a crucial factor in promoting equity in educational settings, consistent with the findings from the existing literature (De Boer et al., 2011; Malak et al., 2018; Knauder and Koschmieder, 2019; Van Mieghem et al., 2020).

The findings of this study also supported Hypothesis 2.2, revealing a significant positive association between pre-service teachers’ perceived behavioral control toward social justice and equity in education (SJPB) construct and their intentions toward social justice and equity in education (SJI) construct (*β* = 0.438, *t* = 4.337, *p* < 0.001). This finding is endorsed by previous research (Knabe, 2009). Perceived behavioral control, a concept closely related to self-efficacy, has been more commonly studied compared to perceived behavioral control when measuring its influence on a person’s intent (Bandura, 1997). Previous studies have also indicated that teachers’ intentions concerning the implementation of inclusive and social justice-based educational practices are significantly predicted by their self-efficacy (Malak et al., 2018; Hellmich et al., 2019). Hence, it is proven in the context of our study that perceived behavioral control plays an essential role in enhancing pre-service teachers’ intentions for the implementation of equitable education policies.

Our results indicated a positive and significant association between pre-service teachers’ subjective norms toward social justice and equity in education (SJSN) and their attitudes (SJA) toward these principles (*β* = 0.434, *t* = 6.185, *p* < 0.001), as well as their perceived behavioral control (SJPB) toward social justice and equity in education (*β* = 0.521, *t* = 9.724, *p* < 0.001). Hence, hypotheses 2.3 and 2.4 were supported. Subjective norms, in the context of social justice and equity in education, refer to the extent to which a teacher’s intention to implement equitable educational practices is influenced by significant others such as school principals, peers, colleagues, and parents of students (Gilor and Katz, 2019). Although perceived norms generally have a weaker influence on intentions compared to attitudes and perceived behavioral control (Armitage and Conner, 2001), in the case of teachers’ implementation of inclusion and equity in education, subjective norms play a significant role. Prior research has shown that subjective norms influence teachers’ attitudes and perceived behavioral control regarding equitable educational practices (Karahanna et al., 2011; Martin et al., 2021). The influence of subjective norms on teachers’ willingness to implement equitable educational practices is further shaped by the level of attitude teachers attach to their opinions (Ajzen, 2020). Teachers who receive positive reinforcement and support from significant others are more likely to develop favorable attitudes toward social justice and equity, leading to a stronger commitment to implementing these principles in their classrooms. These findings highlight the importance of considering subjective norms in promoting social justice and equity in education among pre-service teachers. Understanding and addressing the influences that significant others exert on teachers’ attitudes and perceived behavioral control can enhance teacher preparation programs and professional development initiatives aimed at fostering more inclusive and equitable educational practices. By encouraging a positive and supportive social environment, educators can be better equipped to promote social justice and equity in their classrooms, contributing to a more inclusive educational system overall.

## 7. Conclusion

This study provided empirical evidence to combine the community of inquiry framework with the theory of planned behavior to investigate the association between social media-based COI and pre-service teachers’ intentions concerning social justice and equity in education. The research also explored the presence of pre-service teachers on social media and its connection with their intention regarding social justice and equity. The results revealed that pre-service teachers in Gilgit-Baltistan use social media for educational purposes, but their participation in regular social media-based programs is limited. However, they were actively engaged with social media both on and off campus. Pre-service teachers demonstrated a strong social presence, cognitive presence, and teaching presence within the social media-based COI while contributing to an enriched learning process. Additionally, pre-service teachers demonstrated a significant intention toward promoting social justice and equity in education. It demonstrated their willingness and motivation to advocate for the principles of social justice and equity in education. The findings highlighted a positive association between social media-based COI and pre-service teachers’ intentions toward social justice and equity in education. This study concluded that social media-based COI provides an effective platform for pre-service teachers to enhance their learning experiences. The combination of social presence, teaching presence, and cognitive presence in this context fosters a conducive environment for learners, facilitating their engagement with the important issues of social justice and equity in education.

### 7.1. Practical implications

This study has practical implications for the Higher Education Commission (HEC) of Pakistan, teacher education institutions, and teacher education councils in the country. It would be a valuable approach to utilize social media for developing a community of inquiry for the professional development of both pre-service and in-service teachers. Policymakers can utilize the results to design online lifelong learning programs that would benefit educators in various ways. Designing a community of inquiry through social media would also pave the way for organizing versatile teacher education programs, such as certificate courses, by facilitating the adoption and acceptance of social media-based tools in the curriculum.

We offer several key recommendations based on the study’s results.

First, teacher education programs should actively incorporate social media-based COI as a teaching tool to enhance pre-service teachers’ learning experience. Social media-based COI has the potential to replace traditional teaching methods, which can be especially useful for distance education programs to target remote areas. The use of social media-based COI can be advantageous in remote and distant areas like Gilgit Baltistan, where access to traditional educational resources may be limited.

Second, universities should ensure the integration of social media in education as a mandatory tool to facilitate the teaching-learning process. They should also proactively provide opportunities and encourage educators to adopt social media for educational purposes. Teachers can play a key role by initiating dialog on social media platforms and engaging pre-service teachers in the learning process.

Third, universities should concentrate on enhancing the social presence of pre-service teachers on social media, as this factor is closely associated with teaching and cognitive presence. Educational institutions can develop a conducive environment for learning and knowledge exchange by providing opportunities to build a strong social presence of pre-service teachers in social media-based COI.

Finally, Pakistan can take strides in enhancing the professional development of educators. It may involve promoting innovative approaches to technology adoption by embracing social media-based COI in teacher education programs. Implementing these recommendations can lead to a more progressive and inclusive, social justice-based, and equitable educational landscape in the country.

### 7.2. Limitations of study and future research directions

The generalizability of the results may be limited by certain characteristics of the sample used in this research. Future studies should consider employing larger and more diverse samples to enhance the external validity of the findings and ensure broader applicability. Future research should employ multiple measurement methods, such as self-report measures, observational data, or qualitative approaches, to gain a comprehensive understanding of the constructs under study.

The causality and directionality of the observed relationships cannot be firmly established due to the cross-sectional nature of the research. Future research could employ longitudinal designs to better understand the causal relationships and phenomena among the constructs.

The reliance on self-report data introduces the possibility of response biases, such as social desirability or recall bias. Future studies could employ multiple sources of data collection methods to mitigate potential biases and strengthen the validity of the findings.

The current study has limitations in its scope. It only explored social justice and equity in the educational intentions of pre-service teachers. Thus, further research is required to assess the intention of pre-service teachers toward inclusive education. Lastly, this study has specifically focused on measuring the influence of COI on the intention of pre-service teachers. To gain a comprehensive understanding, future studies should delve into exploring the impact of COI on pre-service teachers’ self-efficacy and emotions, shedding light on additional factors that contribute to their equitable educational intentions.

## Data availability statement

The original contributions presented in the study are included in the article/Supplementary material, further inquiries can be directed to the corresponding author.

## Ethics statement

The study involving human participants was reviewed and approved by the University of Management & Technology, Lahore, Pakistan. The participants provided their written informed consent to participate in this study.

## Author contributions

IU: conceptualization, data curation, formal analysis, methodology, and writing – original draft. MC: conceptualization and validation. EB and MC: methodology and validation. MZA: funding acquisition, conceptualization and supervision. All authors contributed to the article and approved the submitted version.

## Funding

Funds to cover the APC for the publication of this paper were provided by the University of Helsinki.
